# Characterization, Molecular Docking, and In Vitro Dissolution Studies of Solid Dispersions of 20(*S*)-Protopanaxadiol

**DOI:** 10.3390/molecules22020274

**Published:** 2017-02-11

**Authors:** Qi Zhang, Yiqiong Pu, Bing Wang, Yuqin Wang, Tina Tingxia Dong, Tao Guo, Tong Zhang, Zhenzhen Cai

**Affiliations:** 1 Experiment Center for Teaching and Learning, Shanghai University of Traditional Chinese Medicine, Shanghai 201203, China; zhang645654@163.com (Q.Z.); puyiq@163.com (Y.P.); zhangtdmj@hotmail.com (T.Z.); 2 School of Pharmacy, Shanghai University of Traditional Chinese Medicine, Shanghai 201203, China; 3 Zhejiang BioAsia Institute of Life Science, No.1938 Xinqun Road, Economic and Technical Development Zone, Pinghu 314200, Zhejiang, China; wyq@bioasia.com.cn; 4 Division of Life Science and Center for Chinese Medicine, The Hong Kong University of Science and Technology, Hong Kong, China; botina@ust.hk; 5 Center for Drug Delivery Systems, Shanghai Institute of Materia Medica, Chinese Academy of Sciences, Shanghai 201203, China; gotallcn@163.com; 6 Experiment Center for Science and Technology, Shanghai University of Traditional Chinese Medicine, Shanghai 201203, China

**Keywords:** 20(*S*)-protopanaxadiol, solid dispersion, polymer, molecular docking, in vitro dissolution

## Abstract

In this study, we prepared solid dispersions (SDs) of 20(*S*)-protopanaxadiol (PPD) using a melting-solvent method with different polymers, in order to improve the solubility and dissolution performance of drugs with poor water solubility. The SDs were characterized via differential scanning calorimetry (DSC), powder X-ray diffraction (PXRD), Fourier transform infrared spectroscopy (FTIR), nuclear magnetic resonance (NMR), and molecular docking and dynamics study. DSC and PXRD results indicated that PPD crystallinity in SDs was significantly reduced, and that the majority of PPD is amorphous. No interaction was observed between PPD and polymers on FTIR and NMR spectra. Molecular docking and dynamic calculations indicated that the PPD molecule localized to the interpolated charged surface, rather than within the amorphous polymer chain network, which might help prevent PPD crystallization, consequently enhancing the PPD dispersion in polymers. An in vitro dissolution study revealed that the SDs considerably improved the PPD dissolution performance in distilled water containing 0.35% Tween-80 (T-80). Furthermore, among three PPD-SDs formulations, Poloxamer188 (F68) was the most effective in improving the PPD solubility and was even superior to the mixed polymers. Therefore, the SD prepared with F68 as a hydrophilic polymer carrier might be a promising strategy for improving solubility and in vitro dissolution performance. F68-based SD, containing PPD with a melting-solvent preparation method, can be used as a promising, nontoxic, quick-release, and effective intermediate for other pharmaceutical formulations, in order to achieve a more effective drug delivery.

## 1. Introduction

**Figure 1:**
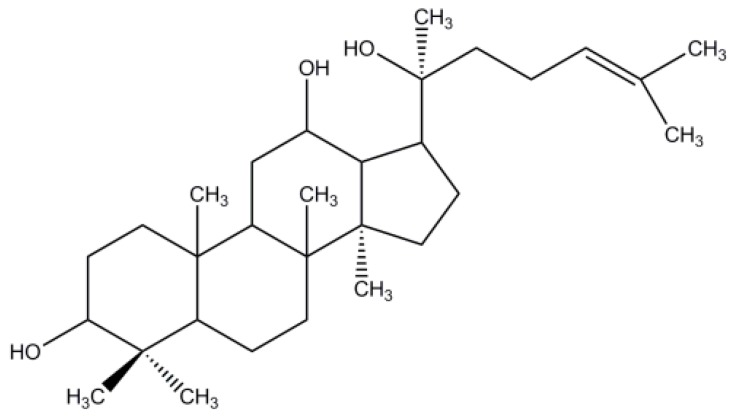
Chemical structure of 20(*S*)-protopanaxadiol (PPD).

Ginseng, the root of *Panax ginseng* C. A. Meyer, has been used as a medicinal plant in China for thousands of years. In modern medicine, ginseng is generally used to treat inflammation, anxiety, depression, fatigue, diabetes, and other disorders [1,2,3,4,5]. Ginsenosides, the main bioactive ingredients [6], can be classified according to the sapogenin structure, as follows: the panaxadiol (e.g., Rb1, Rb2, Rb3, Rc, Rd, Rg3, Rh2, and Rs1), panaxatriol (e.g., Re, Rf, Rg1, Rg2, and Rh1), and oleanolic acid groups (e.g., Ro) [7,8,9]. Ginsenosides exert biological effects, such as anti-inflammation, anti-anxiety, anti-hypertensive, and anti-tumor effects [8,10,11,12,13]. Many effective enzymatic preparations can be used to obtain highly purified 20(*S*)-protopanaxadiol (PPD, aglycon of the panaxadiol group) (Figure 1) [14,15,16,17]. Studies [18,19,20] have reported that PPD is the main metabolite of protopanaxadiol ginsenosides (after oral administration), and its anticancer effect has been demonstrated to be more effective than that of some ginsenosides. Furthermore, the PPD transformation from ginsenosides Rb1, Rb2, and Rc induced an anti-metastatic or anti-carcinogenic effect [21]. Thus, PPD is a potential therapeutic agent for treating various cancers [22,23,24]. Moreover, since PPD has an anti-tumor potential, and an auxiliary anti-tumor agent, the Yijinsheng Capsule (PPD is the major component) has begun late-stage clinical trials. Thus, it is necessary to improve its solubility and dissolution performance.

However, PPD is a chemical biopharmaceutics classification system (BCS) Ⅱ drug with low solubility [25] (approximately 3 μg/mL) and a relatively favorable oil/water partition coefficient [26] (*log P* = 1.72). Researchers reported that this sapogenin exhibited low absolute bioavailability [27], and relative bioavailability of oral formulations (rats: 31%; dogs: 9.6%) [28]. Moreover, its low solubility and first-pass effects [25] led to low bioavailability, which hindered its development and therapeutic applications [29]. Therefore, the development and therapeutic use of this active agent is relatively limited.

Solid dispersion (SD), wherein one or more poorly soluble drugs are dispersed in hydrophilic polymers, is an effective method for improving the solubility and bioavailability of poorly soluble compounds [30,31,32]. Hydrophilic polymer properties determine drug dissolution capacities [33]. SD polymers [34,35,36,37,38,39], such as polyethylene glycol, polyvinylalcohol, cellulose derivatives, polyvinyl pyrrolidone, and poloxamer, are mostly hydrophilic. Three major SD preparation methods include melting [40], solvent [41], and melting-solvent methods [42]. The drug in SD can be dispersed as separate molecules, amorphous particles, or crystalline particles; whereas the carrier can be crystalline or amorphous [43]. Numerous studies have highlighted the advantages of SD in the improvement of the solubility and dissolution rate of drugs with poor water solubility [44,45,46]. For example, SD can convert drugs from a crystalline state to one which is amorphous, thereby avoiding drug recrystallization. Moreover, because of small particle size, the permeability or porosity of SD is relatively high [47,48]. Compared with other techniques for improving the bioavailability of poorly soluble drugs, such as salt formation, particle-size reduction, and solubilization (cosolvent, micelles, and emulsions), SD has several advantages. It reduces the drug particle size to molecular level; whereas other conventional particle-size reduction techniques easily cause agglomeration during formulation, dissolution, or storage [49,50]. Furthermore, SD can be formulated in oral solid preparations, which are more acceptable to patients than liquid products produced through solubilization [51,52]. Therefore, SD has been widely used to develop new drugs as one of the most effective formulations to improve the solubility and bioavailability of water-insoluble drugs [53,54]. Identification of drugs dispersion state in SD is essential. During this quality control process, differential scanning calorimetry (DSC), powder X-ray diffraction (PXRD), Fourier transform infrared spectroscopy (FTIR), and nuclear magnetic resonance (NMR) analyses are commonly used to characterize SD physicochemical formations. DSC and PXRD revealed drug existential states in SDs; whereas FTIR, NMR, and molecular docking calculation are usually employed to study the nature of molecular interactions between drugs and polymer systems.

Up to now, few PPD formulations have been fully developed, among which only a few have improved water solubility, such as SD, emulsions, hydroxypropyl-β-cyclodextrin inclusion, and colloidal dispersions [55,56,57,58,59,60]. In this study, SD with different polymers has been developed via melting-solvent method to optimize formulation and characterize their physicochemical formation with DSC, PXRD, FT-IR, and NMR. A molecular docking and dynamics study also explored PPD and polymer interaction. The water solubility and in vitro dissolution performance of SDs prepared with different formulations were also evaluated.

## 2. Results and Discussion

### 2.1. Drug Loading Efficiency and Solubility Measurement

The drug loading efficiency (DL) of the three SDs (F1–F3) were 8.17% ± 2.93%, 8.13% ± 2.56%, and 7.91% ± 0.74% (*p* > 0.05, all against all SDs), respectively. The solubility of F1, F2, and F3 were 4.66 ± 0.93 μg/mL, 33.24 ± 2.04 μg/mL, and 14.33 ± 0.86 μg/mL, respectively. The solubility of the three formulations (F1–F3) significantly differed from each other (*p* < 0.05).

The solubility measurement results exhibited different polymer effects on the drug dissolvability increase in water. The PPD water solubility in SDs clearly increased when compared with that of pure PPD at 37 °C (approximately 3 μg/mL [25]). F2 exhibited the highest solubility (33.24 ± 2.04 μg/mL), which differed significantly from that of the other SDs (*p* < 0.05).

### 2.2. Characterization

#### 2.2.1. DSC Analysis

**Figure 2:**
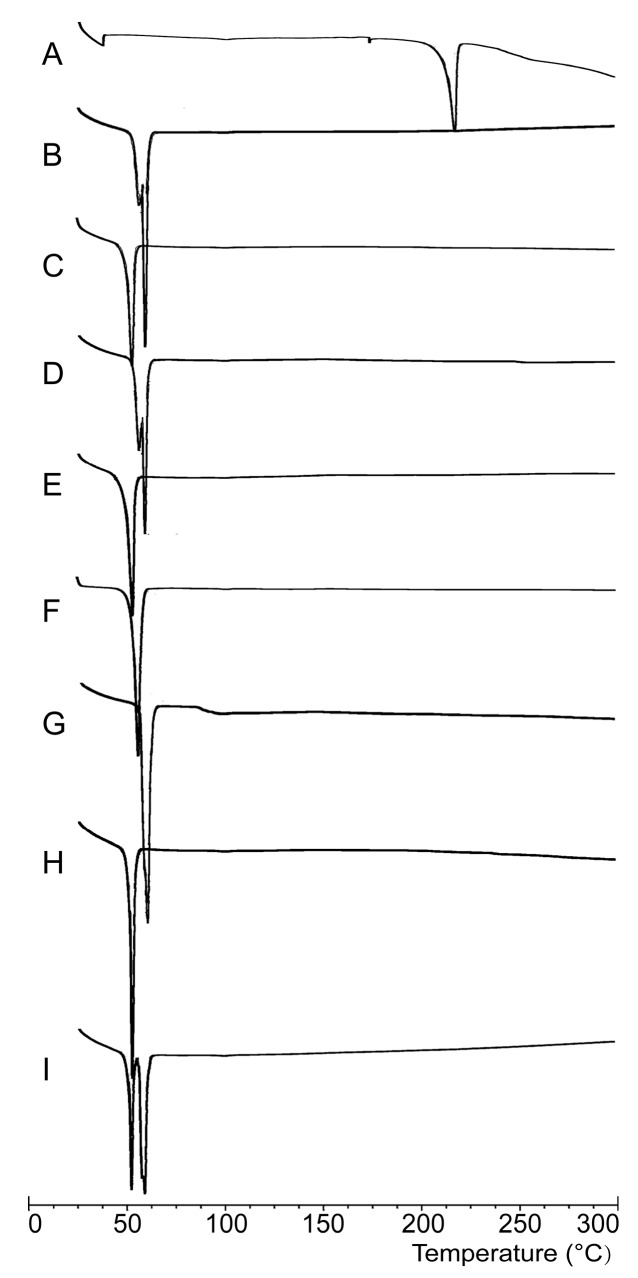
Differential scanning calorimetry (DSC) thermograms of PPD (**A**), Polyethylene glycol 6000 (PEG6000) (**B**), Poloxamer 188 (F68) (**C**), Formulation 1 (F1) (**D**), Formulation 2 (F2) (**E**), Formulation 3 (F3) (**F**), Physical mixture with PEG6000 (PEG6000 PM)(**G**), Physical mixture with F68 (F68 PM) (**H**), Physical mixture with PEG6000 and F68 (PEG6000 and F68 PM) (**I**).

The DSC thermograms of the drug, polymers, SDs, and Physical mixtures (PMs) are shown in Figure 2. The pure PPD exhibited an endothermic peak at 216 °C (Figure 2A), which corresponds to its melting point. PEG6000 (Figure 2B) and F68 (Figure 2C) were detected at their respective melting points of 60 °C and 53 °C, which are the melting points of these polymers. The DSC curves of SDs (Figure 2D–F) and PMs (Figure 2G–I) were similar to those of the polymers, where there was no endothermic peak at 216 °C. PPD existed in SDs in amorphous states. 

In DSC analyses, PPD and polymer characteristic peaks were expected in the DSC thermograms of PMs. However, the DSC thermograms of PMs were quite similar to those of polymers, which can be explained by relating them to the SD formation process and the ratio of PPD and polymers in the formulation. As shown in DSC thermograms, polymer melting points (53 °C and 60 °C) were much lower than that of PPD (216 °C), during DSC analysis, wherein each sample was heated at 10 °C/min from 25 °C to 300 °C. Polymers were melted when the temperature reached 53 °C (F68) or 60 °C (PEG6000). This process was quite similar to SD formation, which indicated that melting PM actually existed in a state of SD. Moreover, large polymer amounts in PM (drug–polymer ratio = 1:10) had masked the existence of PPD DSC analysis, which might also lead to the disappearance of the endothermic peak of PPD in the PM thermograms [61,62,63,64]. PPD was indicated as being in an amorphous state in SDs, wherein no definite melting point of PPD was determined. This inference could also be confirmed by the PXRD analysis results. Another reason for the drug not being detected might be a lack of equipment sensitivity, as reported in [61].

#### 2.2.2. PXRD Analysis

**Figure 3:**
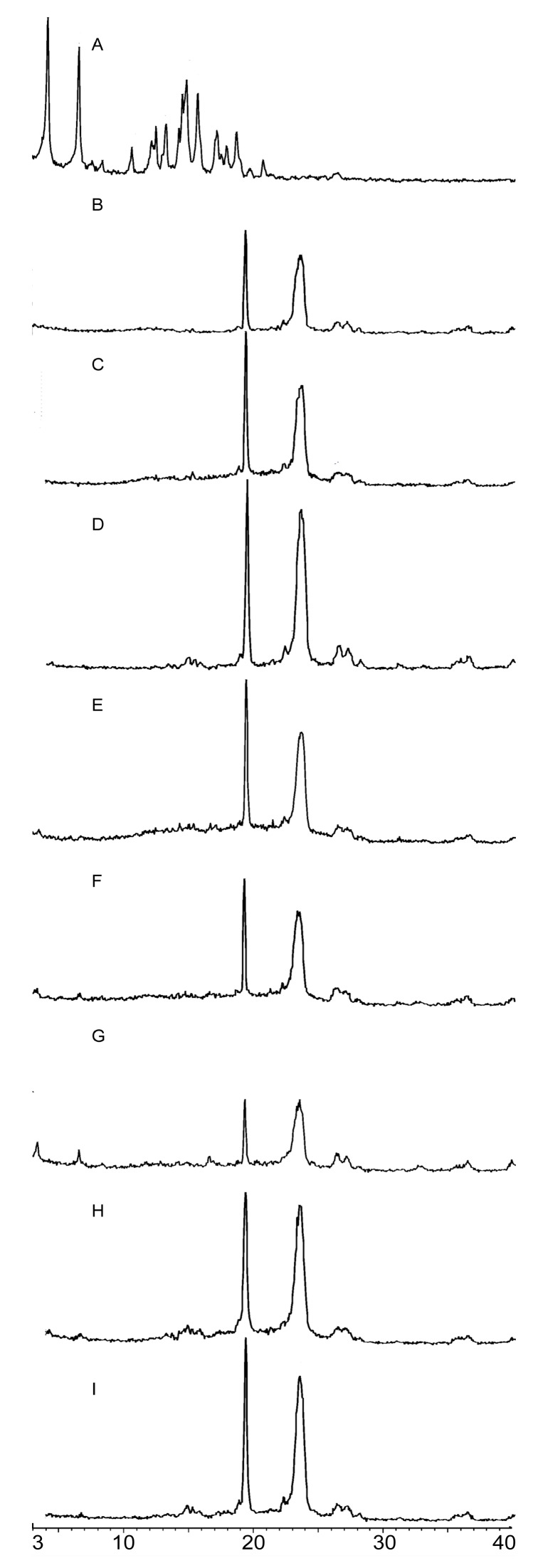
Powder X-ray diffractograms (PXRD) of PPD (**A**), PEG6000 (**B**), F68 (**C**), F1 (**D**), F2 (**E**), F3 (**F**), PEG6000 PM (**G**), F68 PM (**H**), PEG6000 and F68 PM (**I**).

**Figure 4:**
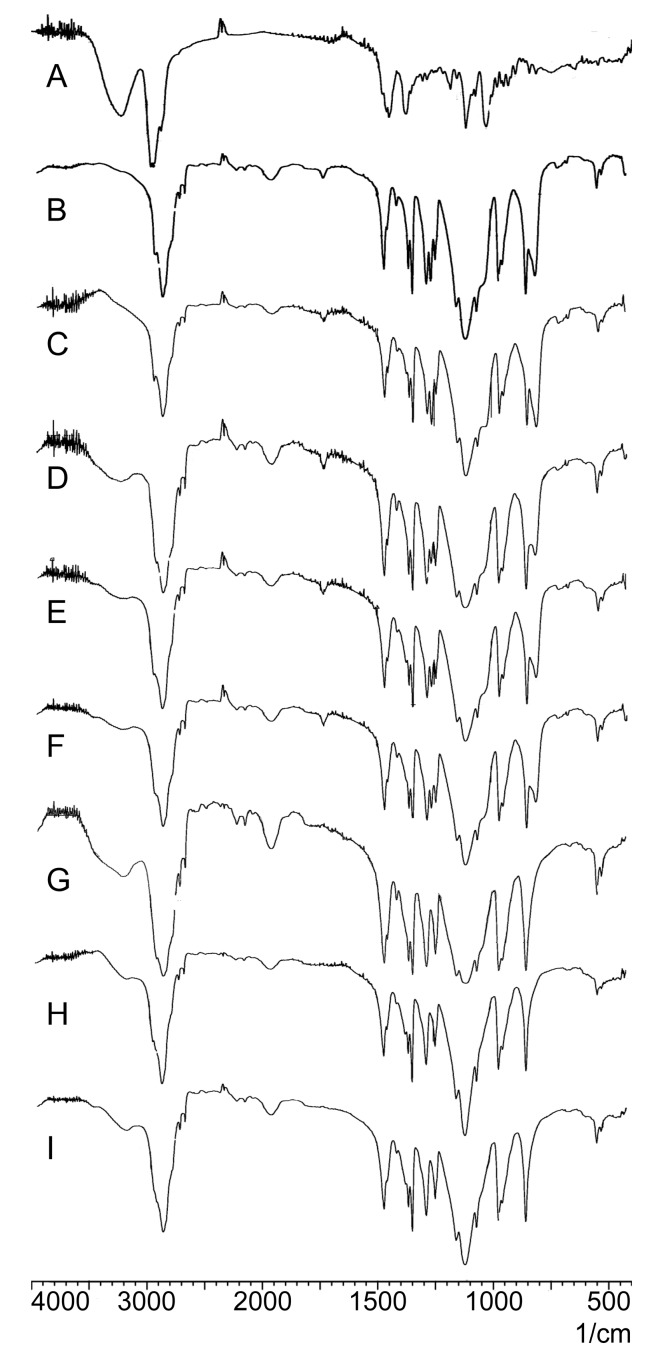
Fourier transform infrared spectroscopy (FTIR) spectra of PPD (**A**), PEG6000 (**B**), F68 (**C**), F1 (**D**), F2 (**E**), F3 (**F**), PEG6000 PM (**G**), F68 PM (**H**), PEG6000 and F68 PM (**I**).

In PPD diffractograms, PPD exhibited high crystallinity, with characteristic crystalline peaks at 4.17°, 6.59°, 14.74°, and 15.68° (Figure 3A), which revealed that PPD was in a crystalline state. PEG6000 (Figure 3B) had characteristic crystalline peaks at 19.34°and 24.48°, whereas F68 (Figure 3C) had characteristic crystalline peaks at 19.25° and 23.57°. SD diffractograms (Figure 3D–F) exhibited characteristic crystalline peaks of the corresponding polymer and some characteristic peak signals of PPD in different degrees of amorphization. F1 and F2 exhibited completely amorphous characters in the absence of PPD characteristic peaks, which indicated that the majority of PPD in these SDs (F1 and F2) presented in the amorphous state (Figure 3D–E). However, some PPD diffraction peaks could be observed in F3 (Figure 3F), which suggested that the majority of PPD in F3 existed in an amorphous state, and only a small amount existed in a crystalline state. PM diffractograms (Figure 4G–I) exhibited polymer characteristic peaks, with slightly stronger signals of PPD than the SD profiles.

PXRD pattern results support the results of solubility measurements presented previously. Amorphous compounds have a less uniform molecular arrangement than crystalline compounds, and this disorder is the driving force for its greater solubility and faster dissolution rates [65,66]. However, some weak characteristic PPD peaks were observed in the PXRD diffractograms of PMs, indicating a polymer influence, which might also be relevant to the preparation method and ratio of the drug and polymers in SD formulation, as mentioned above. However, weaker PPD characteristic peaks were observed in SD diffractograms. This finding indicated that the majority of PPD might be present in an amorphous state in SDs, and a crystalline transformation of PPD is present in the SD formation. The preparation method of solid dispersion was critical in determining the X-ray diffraction pattern. Some reports [61] found that this phenomenon was attributed to either drug conversion to a different polymorph (fusion method) or a conversion to the amorphous state or molecular solution (solvent method). 

#### 2.2.3. FTIR Analysis

As shown in Figure 4A, the FTIR spectra of PPD revealed characteristic peaks at 3221 (-O-H stretching), 2943, 2873 (-C-H stretching), 1120, and 1031 cm^−1^ (-C-O stretching). PEG6000 and F68 exhibited similar constitutional units (-CH_2_CH_2_O-); therefore, their FTIR spectra (Figure 4B,C) were similar. PEG6000 (Figure 4B) and F68 (Figure 4C) exhibited characteristic peaks close to 2890, 1465, and 1110 cm^−1^. Characteristic PPD and polymer peaks were observed in the SD spectra (Figure 4D–F) and PMs (Figure 4G–I).

**Figure 5:**
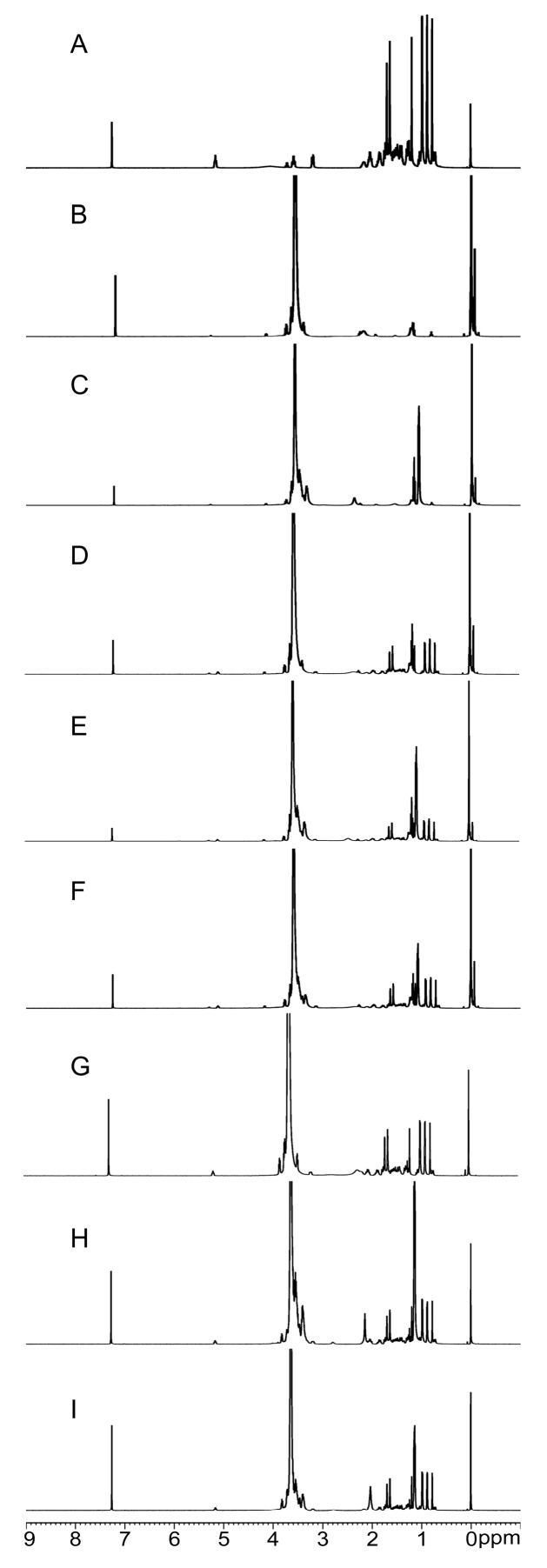
Nuclear magnetic resonance (NMR) spectra of PPD (**A**), PEG6000 (**B**), F68 (**C**), F1 (**D**), F2 (**E**), F3 (**F**), PEG6000 PM (**G**), F68 PM (**H**), PEG6000 and F68 PM (**I**).

FTIR spectroscopy was used to investigate drug and polymer interactions, which may lead to peak broadening and the bathochromic shift of the absorption bands of interacting functional groups. By comparing the PPD and polymer spectra with those of SDs and PMs (Figure 5), the characteristic PPD and polymer peaks could be identified in SD and PM spectrograms, despite the disappearance of some characteristic peaks. This peak disappearance might be associated with the drug-polymer ratio (1:10). Hence, the existence of polymers might conceal some characteristic PPD peaks. The spectrograms exhibited no new functional groups; moreover, it indicated the lack of chemical reactions between PPD and polymers in SDs [67,68].

#### 2.2.4. NMR Analysis

Various feature peaks were observed in the PPD spectra (Figure 5A). A total of eight Hydrogen (H) signals corresponding to eight PPD methyl groups within the range 0.85–1.62 ppm were observed. The H signal at 5.29 ppm represented the PPD olefinic carbon. PEG6000 and F68 signals (Figure 5B,C) almost eliminated the interference in SDs (Figure 5D–F) and PMs (Figure 5G–I), thereby confirming that no new functional groups were present.

^1^H-NMR spectra were used to describe the interactions between PPD and PEG, F68, or in both SDs. A comparison of the ^1^H-NMR spectra of PPD and polymers with SDs and PMs revealed characteristic PPD peaks in the SD and PM spectra, without forming new characteristic peaks. Furthermore, the position of the characteristic PPD peaks did not change, which suggested lack of interactions between PPD and PEG, F68, or in both SDs [69].

### 2.3. Molecular Docking and Dynamics Calculation

**Figure 6:**
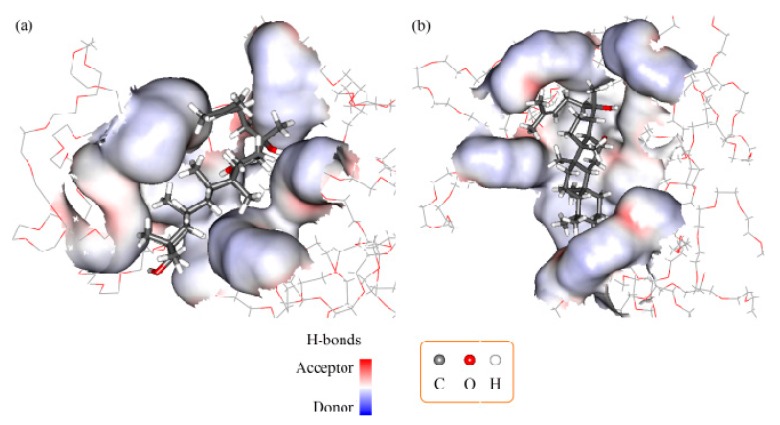
3D interaction conformations of PPD-F68 in F1 (**a**) and PPD-PEG6000 in F2 (**b**).

A computational simulation was used to investigate PPD-PEG6000 (F1), PPD-F68 (F2), and PPD-PEG6000-F68 (F3) interactions at the molecular level. Optimized PPD and F68 conformations were initially produced from linear structures. F1 and F2 simulation results (Figure 6a,b) revealed that the PPD molecule localized to the interpolated charged surface of PEG6000 (or F68) rather than within the amorphous polymer chain network. 

**Figure 7:**
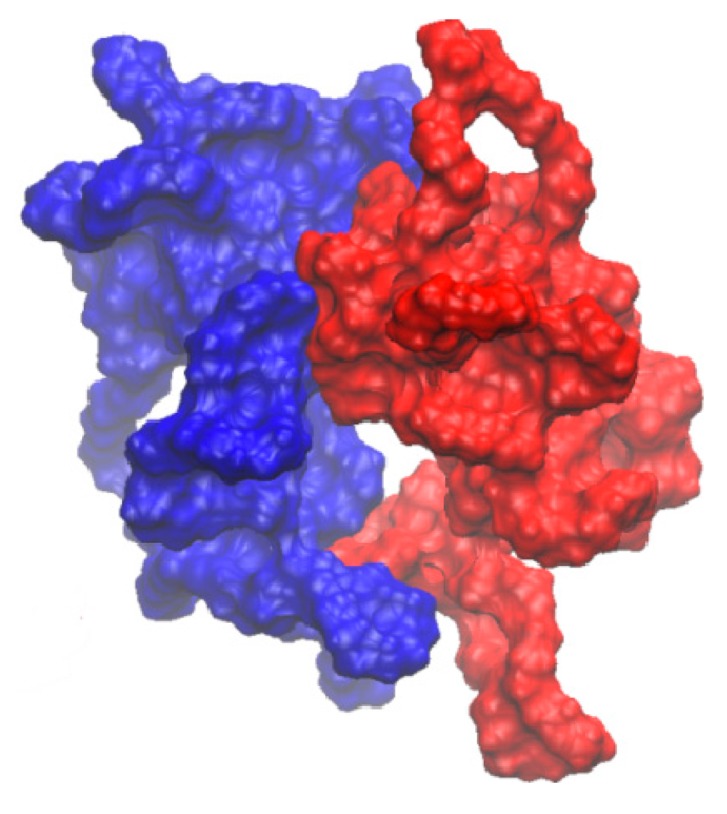
3D interaction conformations of F68 (Blue) and PEG6000 (Red).

The docking-free energy of PPD-PEG6000 (F1) and PPD-F68 (F2) were −3.5 and −4.5 kcal·mol^−1^, respectively. These negative values showed that PEG (or F68) could interact with PPD to prevent PPD crystal formation. This was consistent with the conclusions of the PXRD and DSC methods. The composite ratio of PPD and PEG6000 (or F68) can be considered as 2:1 according to the experimental data. Therefore, in the molecular docking protocol, two PPD and one PEG6000 (or F68) molecules were used. According to this composite ratio, the equilibrium constants of the PPD and F68 interaction should be greater than that between PPD and PEG6000. The computational result (docking-free energy of PPD-F68 in F2 was lower than that of PPD-PEG6000 in F1) was consistent with this estimation of their interaction. Therefore, we believe that an interaction between PEG6000 and F68 occurred (the interaction energy is −442 kcal·mol^−1^). These interactions might lead to slight decrease in the PPD-PEG-F68 (F3) drug loading efficiency when compared with PPD-PEG6000 (F1) and PPD-F68 (F2), without any statistical difference (*p* > 0.05), as shown in the DL determination results. Figure 7 shows the most probable interaction conformation between PEG6000 and F68 in F3. However, due to the low concentrations of PEG6000 and F68 in F3, any interaction between them was not very obvious.

The molecular docking and dynamic study results suggested that the PPD molecule localized to the interpolated charged surface of PEG6000 (or F68), rather than within the amorphous polymer chain network. PEG6000 (or F68) might interact with PPD in order to prevent PPD crystal formation, which helped enhance the PPD dispersity in the polymers. The interaction between PEG6000 and F68 in F3 might lead to a lower drug loading efficiency. However, the effect was not obvious due to the low PEG6000 and F68 concentrations in F3.

### 2.4. In Vitro Dissolution Study

**Figure 8:**
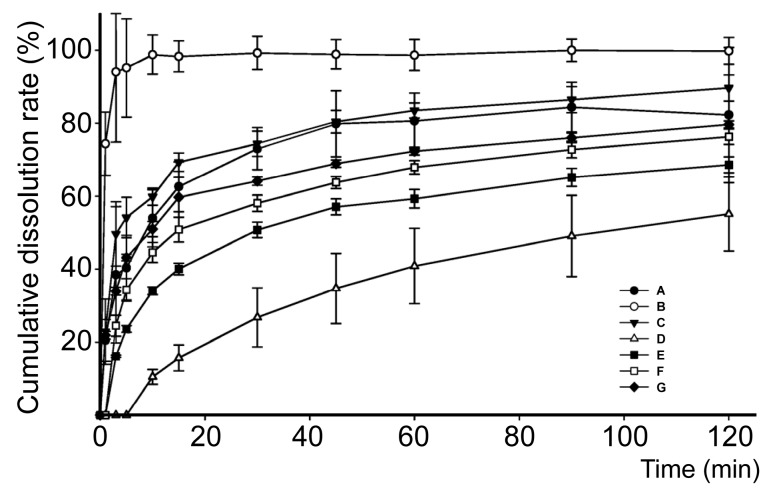
Dissolution profiles of F1 (**A**), F2 (**B**), F3 (**C**), PPD (**D**), PEG6000 PM (**E**), F68 PM (**F**), PEG6000 and F68 PM (**G**).

The in vitro dissolution behaviors of PPD, SDs, and PMs in the dissolution media under sink conditions are shown in Figure 8. Pure PPD had the slowest and lowest dissolution rate, whereas F2 had the fastest and highest dissolution rate. In the first 15 min, each SD exhibited a 60% dissolution rate, whereas PPD only exhibited a 15% rate. After 60 min, the SD dissolution profiles gradually increased to 80%. After 120 min, among all of the dissolution profiles, F2 had the highest rate at approximately 99.8%. F3 and F1 exhibited the second highest rate at approximately 89.6% and 82.2%, respectively, whereas the dissolution rate of the pure drug was approximately 55.1%. The dissolution speed and rate of the PMs were higher than those of pure PPD, but lower than those of the SDs. The final dissolution rates of all of the SDs significantly differed from that of pure PPD (*p* < 0.05). Furthermore, the F2 dissolution rate significantly differed from that of F1 (*p* < 0.05).

The in vitro dissolution performance was measured to evaluate SD effects on promoting in vitro dissolution. The dissolution profile and statistical analyses revealed that F68 was a more suitable SD polymer than PEG6000 for the PPD-SDs formulations. SDs had a higher dissolution rate than PMs. The in vitro dissolution study results suggested that the F68 exerted superiorly favorable effects on the dissolution rate of PPD, whose effects might be related to the solubilization of these hydrophilic polymers. This might contribute to the development of new PPD agents and other poorly soluble drugs [70].

## 3. Materials and Methods

### 3.1. Materials

20(*S*)-protopanaxadiol (purity > 98%, No. 111747-200501, National Institutes for Food and Drug Control, Beijing, China), PEG6000 (Sinopharm Chemical Reagent Co., Ltd., Shanghai, China), poloxamer 188 (F68, BASF, Germany), and Tween^®^-80 (T-80, Sinopharm Chemical Reagent Co., Ltd., Shanghai, China) were used in the study. The acetonitrile was of chromatographic grade and purchased from Merck Co. Ltd. Other solvents were of analytical grade.

### 3.2. Formulation Design

SD formulations were composed of PPD (purity > 98%), T-80, and polymers (PEG6000, F68, and PEG6000 and F68 mixture). The formulations are detailed in Table 1.

### 3.3. SD Preparation

SDs were prepared via a melting-solvent method at a mass ratio of 1:10 (drug:polymer). PPD was completely dissolved in the proper amount of absolute alcohol containing T-80 (PPD:absolute alcohol:T-80 = 1:8:0.8, *m*/*v*/*m*). Subsequently, the solution was transferred into the melting matrix (polymer) in a 80 °C water bath and stirred for 15 min. The resultant was frozen at −20 °C for 24 h, porphyrized by an agate mortar and pestle, and stored in a desiccator at ambient temperature.

### 3.4. Physical Mixture Preparation

PMs were prepared by the simple intensive mixing of PPD and PEG6000 and/or F68 at certain ratios (1:10, 1:10, and 1:5:5, *m*/*m*) in a mortar until homogeneous. Resultant mixtures were sieved and then kept in a desiccator at room temperature until further study.

### 3.5. Determination of Drug Loading Efficiency 

The PPD content in the samples was determined through high-performance liquid chromatography (HPLC) using an Agilent 1200 system (Agilent Technologies, Santa Clara, CA, USA) equipped with a UV detector and it was detected at a wavelength of 203 nm. The separation was performed using a XB-C18 column (Welch, Shanghai, China; 5 μm, 4.6 mm × 250 mm). The mobile phase was composed of acetonitrile and deionized water (88:12, *v*/*v*) and eluted at a rate of 1.0 mL/min. The column temperature was maintained at 25 °C. The drug loading (DL) efficiency was measured as follows. After the prepared SD was completely dissolved in methanol, the supernatant was withdrawn and filtered through a membrane filter (0.45 μm). The amount of PPD in the resulting solution was quantitatively analyzed using HPLC. Each sample was measured in triplicate. Lastly, the DL was calculated using the following equation:

(1) $$\mathrm{DL}\left(\%\right)=\frac{\text{Amount of PPD in SDs}}{\text{Amount of SDs}}\times 100\%$$

### 3.6. Characterization of SDs

#### 3.6.1. DSC Analysis

Physicochemical properties of PPD in SDs were evaluated through DSC. Before testing, the solid sample was dried at 40 °C to remove residual water. PPD, SDs, and PMs were analyzed under a nitrogen atmosphere through DSC (DSC-Q2000, TA Instruments, New Castle, DE, USA), and each sample was heated at 10 °C/min from 25 °C to 300 °C.

#### 3.6.2. PXRD Analysis

PXRD patterns were evaluated via PXRD (Bruker D8 Advance). Samples were exposed to Cu radiation under 40 mA and 40 kV. The scanning angle (*2θ*) ranged from 3° to 40° at 1°/s.

#### 3.6.3. FTIR Analysis

FTIR was performed using a Fourier transform infrared spectrophotometer (FTIR Presitage 21 Fourier Transform Infrared Spectrophotometer, Shimadzu). Potassium bromide discs were prepared by mixing a small amount of the sample with potassium bromide and compressing the mixture. The frequency ranged from 4000 cm^−1^ to 400 cm^−1^.

#### 3.6.4. Nuclear Magnetic Resonance

The ^1^H-NMR spectra [71] for SDs were analyzed by an AVANCE III 400 MHz UltraShield-PlusTM (Bruker, Karlsruhe, Germany) NMR spectrometer using deuterated chloroform (CDCl_3_) as the solvent.

### 3.7. Molecular Docking and Dynamics Calculation

PPD molecular structure was extracted from the single crystal structure of 20-*O*-β-glucopyranosyl-20(*S*)-protopanaxadiol dehydrate obtained from Cambridge Structural Database (Refcode: JEPJEU) and reconstructed. PEG6000 and F68 molecular structures were constructed manually. The molecular dynamics program Amber 12 (University of California, San Francisco, CA, USA) was used for energy minimization and molecular dynamics calculation. AmberTools in Amber 12 was used to prepare Amber starting stuffs. Partial atomic charges of PEG6000 or F68 units were calculated via Gaussian 09 package. The docking program AutoDock Vina 1.1.2 (The Scripps Research Institute, San Diego, CA, USA) [72] was used to perform an automated molecular docking calculation.

Firstly, energy minimization protocol was executed to prepare PPD, PEG6000, and F68 models. Then, a 20 ns molecular dynamics simulation was run for PEG6000 and F68. Following this, the 10 highest scoring conformation with one or two PPD molecules separately docked to PEG6000 or F68 molecule were generated. Finally, one conformation with relatively low energy was used for the optimization of the successive structure.

During the minimization protocol, all models were placed under vacuum. Optimized PEG6000 and F68 molecules were put into separated water boxes, which had a 10 Å width. Then, a 20 ns molecular dynamics simulation was performed. A non-bond cutoff distance of 18.5 Å, spline width of 1.0 Å, and buffer width of 0.5 Å were used. During the docking protocol, a Lamarckian Genetic Algorithm (LGA) combined with a grid-based energy evaluation method were used for pre-calculating grid maps according to the interatomic potentials of all of the atom types present in the host and guest molecules, including Lennard-Jones potentials for van der Waals interactions and Coulomb potentials for electrostatic interactions. A grid map of dimensions 80 Å × 80 Å × 80 Å, with a grid spacing of 0.375 Å, was used to cover the F68 or PEG6000 structure. Using AutoDockTools (Version 1.4.5, The Scripps Research Institute, La Jolla, CA, USA), the atomic partial charges were calculated using the Gasteiger-Marsili method [73]. Parameters used for the global search included an initial population of 50 individuals, with a maximal number of energy evaluations of 1,500,000, and a maximal number of generations of 50,000 as an end criterion. The Autodock Vina scoring function, which is based on experience, and other docking parameters were set to default.

### 3.8. Solubility Measurements

The water solubility was determined by a modified shake-flask method [74,75]. Excess SDs were dispersed in 5 mL distilled water and shaken at 100 r/min and 37 °C for 24 h. Samples were then centrifuged at 12,000 r/min for 5 min. The resultant supernatant was withdrawn and filtered using a 0.45 μm membrane. The PPD content in the saturated solution was determined by the HPLC method, as previously described.

### 3.9. In Vitro Dissolution Study

The in vitro dissolution studies of PPD, SDs, and PMs were performed using a Chinese Pharmacopoeia (2015) type III dissolution apparatus [70,76,77]. The dissolution media was comprised of 200 mL distilled water, containing 0.35% T-80 at 37 ± 0.5 °C. The stirring speed was 100 r/min. PMs and SDs of the formulations, containing approximately equivalent amounts of 8 mg PPD, were immersed into the dissolution media. Sample solutions of 2.0 mL were withdrawn at specific time intervals and filtered with a 0.45 μm membrane for sample analysis. Furthermore, preheated fresh media of an equal volume was added for compensation at every withdrawal. PPD concentration in the samples was determined by HPLC, as previously described, and the cumulative drug dissolution rate at each time point was profiled. Each experiment was performed in triplicate.

### 3.10. Statistical Analysis

All data are presented as mean ± standard deviation or mean. The statistical significance of the differences was determined using ANOVA. In all tests, *p* < 0.05 was considered significant. Data analysis was performed with the SPSS software package (Version 19.0, IBM, NY, USA).

## 4. Conclusions

In this study, SDs were prepared with different polymers, and SD formations were characterized via different analytical methods. DSC and PXRD analyses results indicated that SD crystallinity reduced significantly, whereas the majority of PPD in SDs existed in an amorphous state. Furthermore, no interaction was observed between the PPD and polymers on FTIR and NMR spectra. Molecular docking and dynamic calculations indicated that the PPD molecule localized to the interpolated charged surface of PEG6000 (or F68) rather than within the amorphous polymer chain network, which might have helped prevent PPD crystal formation consequently enhancing the PPD dispersity in the polymers. An in vitro dissolution study revealed that, compared with pure PPD, SDs considerably improved the dissolution performance in distilled water (0.35% T-80). Furthermore, among three SD formulations, F68 was the most effective in improving the PPD solubility and was superior to F68 and PEG6000 (1:1).

Consequently, this study revealed the potential of SDs to increase PPD solubility and dissolution performance. As a pharmaceutical intermediate, SDs can be further processed into tablets [78], capsules [79], pills [80], and other formulations. However, in vivo studies on PPD are needed for further comprehensive evaluation of its SD products.

## Figures and Tables

**Table 1:** SD formulation ratio (*m*/*m*) of PPD with different polymers.

Formulation	PPD (g)	Polymer (g)		Tween-80 (g)
		PEG6000	F68	
F1	0.5	5.0	-	0.4
F2	0.5	-	5.0	0.4
F3	0.5	2.5	2.5	0.4
